# Laboratory Mice – A Driving Force in Immunopathology and Immunotherapy Studies of Human Multiple Myeloma

**DOI:** 10.3389/fimmu.2021.667054

**Published:** 2021-06-02

**Authors:** Michael Pisano, Yan Cheng, Fumou Sun, Binod Dhakal, Anita D’Souza, Saurabh Chhabra, Jennifer M. Knight, Sridhar Rao, Fenghuang Zhan, Parameswaran Hari, Siegfried Janz

**Affiliations:** ^1^ Division of Hematology and Oncology, Department of Medicine, Medical College of Wisconsin, Milwaukee, WI, United States; ^2^ Interdisciplinary Graduate Program in Immunology, University of Iowa, Iowa City, IA, United States; ^3^ Departments of Psychiatry, Medicine, and Microbiology & Immunology, Medical College of Wisconsin, Milwaukee, WI, United States; ^4^ Division of Hematology, Oncology and Marrow Transplant, Department of Pediatrics, Medical College of Wisconsin, Milwaukee, WI, United States; ^5^ Blood Research Institute, Versiti Wisconsin, Milwaukee, WI, United States; ^6^ Myeloma Center, Department of Internal Medicine and Winthrop P. Rockefeller Cancer Institute, University of Arkansas for Medical Sciences, Little Rock, AR, United States

**Keywords:** genetically engineered mouse models of human cancer, auto- and xenografting, immune, immunodeficient mice models, immune pathogenesis, Myeloma

## Abstract

Mouse models of human cancer provide an important research tool for elucidating the natural history of neoplastic growth and developing new treatment and prevention approaches. This is particularly true for multiple myeloma (MM), a common and largely incurable neoplasm of post-germinal center, immunoglobulin-producing B lymphocytes, called plasma cells, that reside in the hematopoietic bone marrow (BM) and cause osteolytic lesions and kidney failure among other forms of end-organ damage. The most widely used mouse models used to aid drug and immunotherapy development rely on *in vivo* propagation of human myeloma cells in immunodeficient hosts (xenografting) or myeloma-like mouse plasma cells in immunocompetent hosts (autografting). Both strategies have made and continue to make valuable contributions to preclinical myeloma, including immune research, yet are ill-suited for studies on tumor development (oncogenesis). Genetically engineered mouse models (GEMMs), such as the widely known Vκ*MYC, may overcome this shortcoming because plasma cell tumors (PCTs) develop *de novo* (spontaneously) in a highly predictable fashion and accurately recapitulate many hallmarks of human myeloma. Moreover, PCTs arise in an intact organism able to mount a complete innate and adaptive immune response and tumor development reproduces the natural course of human myelomagenesis, beginning with monoclonal gammopathy of undetermined significance (MGUS), progressing to smoldering myeloma (SMM), and eventually transitioning to frank neoplasia. Here we review the utility of transplantation-based and transgenic mouse models of human MM for research on immunopathology and -therapy of plasma cell malignancies, discuss strengths and weaknesses of different experimental approaches, and outline opportunities for closing knowledge gaps, improving the outcome of patients with myeloma, and working towards a cure.

## Introduction

Multiple myeloma (MM) is a neoplasm of terminally differentiated, post-germinal center, immunoglobulin (Ig)-producing B-lymphocytes, called plasma cells, that reside in the hematopoietic bone marrow (BM). Quintessential disease manifestations include a serum M-spike (monoclonal Ig, paraprotein) and signs of end-organ damage known as CRAB symptoms: hypercalcemia, renal insufficiency, anemia, and lytic bone lesions (1). The most recent estimate of the US National Cancer Institute SEER (Surveillance, Epidemiology, and End Results) Program predicts slightly more than 32 thousand cases of newly diagnosed myeloma (NDMM) and nearly 13 thousand disease-specific deaths in 2020. This renders MM the second most common and one of the deadliest blood cancers in the United States. Owing to newly developed myeloma agents, particularly proteasome inhibitors (PIs), immunomodulatory drugs (IMiDs) and monoclonal antibodies (mAbs), the outcome for patients with MM has significantly improved in recent years (2), making it possible, at long last, to cure a tangible number of patients (3). However, in the great majority of cases, following a period of successful therapy, myeloma relapses as a drug-refractory, aggressive disease that leaves few, if any, therapeutic options. The root causes of progression to relapsed and/or therapy-refractory myeloma (RRMM) include tumor cell-intrinsic changes such as point mutations in drug response genes (4), copy number alterations that may abrogate tumor suppressor pathways (5), epigenomic aberrations modifying gene expression (6), and increased cancer stemness, which may impact lineage fidelity and tumor dormancy to name but two changes (7). An equally important yet tumor cell-extrinsic driver of RRMM pathophysiology is the tumor microenvironment (TME), which provides myeloma-promoting interactions with resident BM cells, including specimens of the innate and adaptive immune system (8). Enhanced understanding of immune regulation of the BM microenvironment (BMME) has not only shed light on pathways of myeloma progression but also greatly advanced myeloma treatment over the past decade (9).

**Figure 1:**
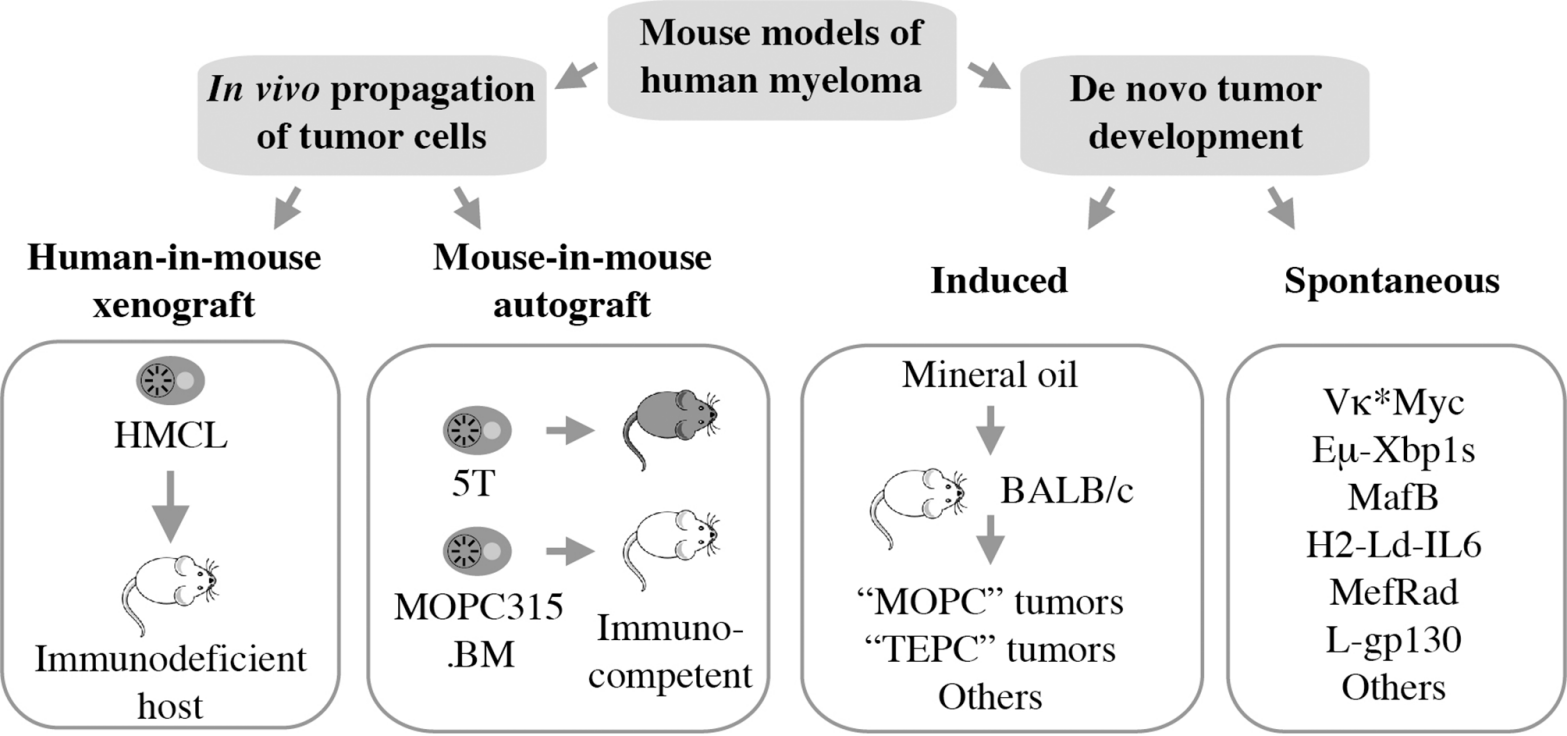
Mouse models of human myeloma. Xeno- and autografting relies on *in vivo* propagation of fully transformed tumor cells. Models of tumor development include peritoneal plasmacytomas that can be readily induced in genetically susceptible BALB/c mice and myeloma-like tumors that arise spontaneously in a variety of transgenic mice.

Mouse models of human myeloma have provided preclinical research tools for elucidating the role of the immune system in the natural history of plasma cell neoplasia and in assessing candidate immunotherapies for myeloma (10, 11). Numerous experimental model systems are available, however, none perfectly replicate human myeloma (**Figure 1**). The most widely used models rely on *in vivo* propagation of human myeloma cells in immunodeficient hosts (human-in-mouse xenografting) (12–14) or myeloma-like plasma cells from C57BL/6 (B6) (15) or BALB/c (C) mice (16) in genetically compatible (syngeneic) immunocompetent hosts (mouse-in-mouse autografting). Both strategies have made and continue to make important contributions to myeloma research (17, 18), but are hampered by the reality that *in vivo* transfer of malignant cells (tumor transplantation) is not suitable for studying tumor development (oncogenesis). In other words, xeno- and autografting of neoplastic plasma cells bypasses the natural course of human myelomagenesis that begins with monoclonal gammopathy of undetermined significance (MGUS) (19), progresses to smoldering myeloma (SMM) (20), and eventually transitions to frank neoplasia (NDMM). Laboratory mice, in which plasma cell tumors (PCT) arise *de novo* (spontaneously) in a fully immunocompetent microenvironment, may remedy this shortcoming yet are undermined by other limitations, including complex breeding schemes, cost and time. Here we review the contribution of mouse models to advances in immunopathology and -therapy of human myeloma, discuss strengths and weaknesses of different experimental approaches, and outline opportunities for closing knowledge gaps.

## Immunopathology And -Therapy Of Multiple Myeloma

### Immune Editing and Immune Dysfunction in Myeloma

**Figure 2:**
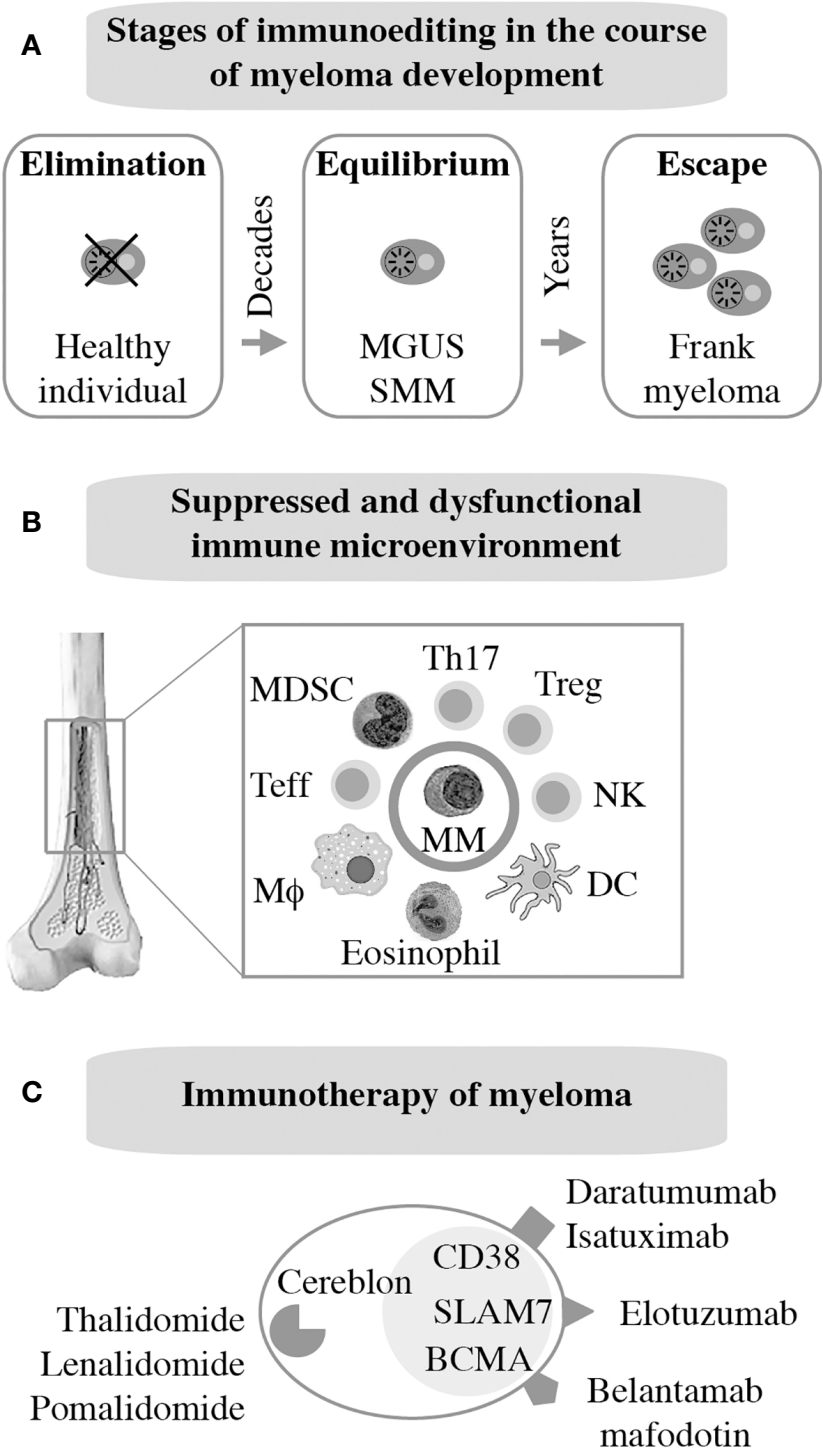
Immunopathology and -therapy of myeloma. Evidence indicates that myeloma development is promoted, in part, by the gradual breakdown of immunosurveillance **(A)**. Consequently, patients with myeloma have a suppressed and dysfunctional immune microenvironment **(B)**. 2 subsets of Tregs that discriminate MGUS from MM (31); 2 subsets of terminal effector T cells (T_TE_) are involved in MGUS to MM transition (32); Attrition of BM-resident T cells due to loss of “stem-like” TCF1/7hi T cells may underlie loss of immune surveillance in myeloma (33). Enhanced understanding of this microenvironment has been key for the development of immunotherapies of myeloma **(C)**.

As mentioned above, frank myeloma is invariably preceded by the precursor conditions MGUS (19) and SMM (21). MGUS is, in most cases, asymptomatic (22) and is usually detected years before frank MM manifests. MGUS progresses to active myeloma at the slow and constant rate of approximately 1% per year (21, 23). Consistent with the more advanced stage of tumor development, the progression rate of SMM is higher: 10% per annum in the first 5 years and 3% thereafter (24). Notably, BM plasma cells of individuals with MGUS exhibit a gene expression profile that is highly similar to that of myeloma (25) and MGUS plasma cells contain many of the cytogenetic changes (chromosomal translocations, gains and deletions) typically seen in myeloma cells (26–28). These findings have long raised suspicion that MGUS may in fact be a malignancy that is suppressed by a strong, extrinsic force, such as a cognate cytotoxic immune response (immune surveillance). Because most cases of MGUS do not progress to MM, this surveillance mechanism must be effective and enduring, essentially covering the entire lifespan of most individuals harboring an aberrant plasma cell clone of this sort. A large body of recent work expertly reviewed elsewhere (29, 30) strongly suggests that the breakdown of immunologic surveillance is at the heart of the MGUS-to-MM transition. According to this theory, known as cancer immunoediting (**Figure 2A**), the immune system is initially highly successful in eliminating abnormal plasma cells (Elimination stage), but then switches to an impasse that permits a limited number of these cells to survive in a quiescent or dormant state (Equilibrium) that may last for many years in individuals with MGUS (34). For reasons that are not yet clear, the equilibrium is eventually disrupted in a subset of patients, allowing the aberrant cell clone to evade immune control (Escape) and fuel the malignant growth that underlies active myeloma. In sync with that scenario, immune suppression caused by regulatory T cells, myeloid derived suppressor cells (MDSC) and dysfunctional effector T lymphocytes, is a hallmark of NDMM (**Figure 2B**). Growth and survival support of myeloma cells by innate immune cells, including conventional and plasmacytoid dendritic cells (35, 36) and eosinophils (37), is the flip side of the same coin. Of practical relevance for patient care is the knowledge that immune dysfunction in patients with myeloma may lead to increased risk of infections (38) and lack of a vigorous vaccination response (39) – something to be mindful about in the midst of SARS-CoV-2 (40).

### Immunotherapy of Myeloma

The past decade has witnessed tremendous progress in immunotherapeutic approaches to myeloma. Authoritative and up-to-date reviews are available (29, 41). Current FDA-approved interventions include monoclonal antibodies targeting CD38 [daratumumab (42), isatuximab (43)] or SLAMF7 [elotuzumab (44)] on the surface of tumor cells. An antibody-drug conjugate (ADC) that binds to BCMA [belantamab mafodotin (45)] has also been approved just recently. Additional BCMA-targeted therapies, in particular chimeric antigen receptor (CAR) T cells and bispecific T cell engagers (BITEs), are in advanced stages of clinical development. Immune modulation using small-drug inhibitors of cereblon, a component of an E2 ubiquitin ligase complex, is also approved for treatment of myeloma and is widely used for maintenance therapy internationally. Immunomodulatory drugs of this sort, dubbed IMIDs, include thalidomide (46), lenalidomide (47, 48), and pomalidomide (49). Next-generation cereblon-targeting agents that promise to overcome acquired resistance to IMIDs are in clinical trial (50). **Figure 2C** provides an overview of myeloma immunotherapies in clinical use. Not shown are proteasome inhibitors (PIs), a class of targeted myeloma agents that, in the past, have not been associated with immune-mediated myeloma-inhibiting effects. However, recent work demonstrates that modulating the immune microenvironment of myeloma, by virtue of inducing immunogenic cell death (51), primes a cytotoxic immune response to tumor cells, thereby contributing to disease control in a proteasome-independent manner.

## Xenograft Models Of Myeloma

### Propagating Human Myeloma Cell Lines (HMCLs) in NSG and NRG Hosts

**Figure 3:**
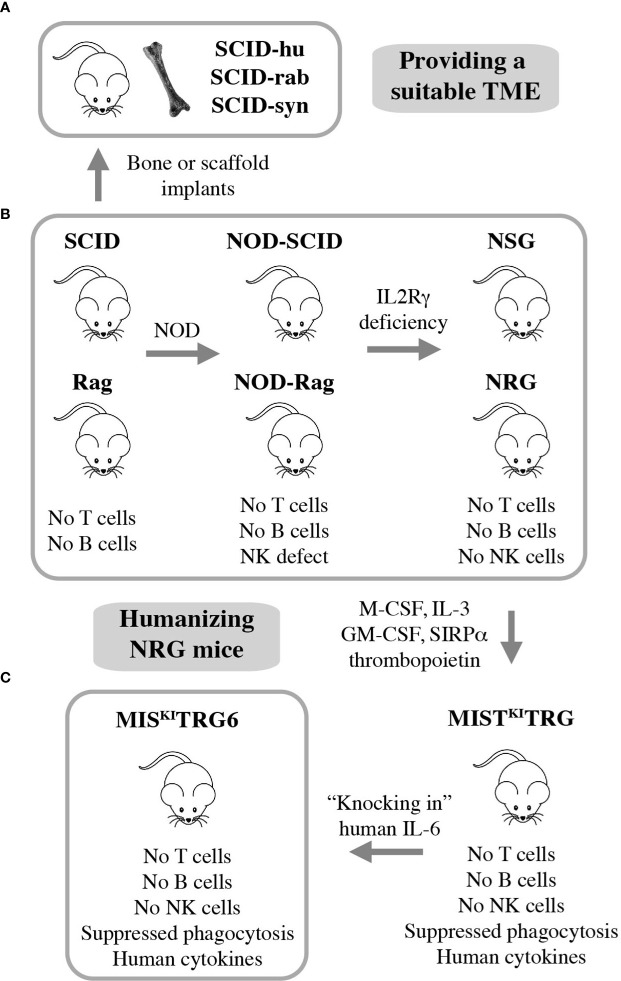
Xenografting human myeloma in immunodeficient mice. NSG and NRG mice are widely employed for preclinical studies using human myeloma cell lines (HMCLs) but are limited in terms of hosting primary, patient-derived myeloma cells **(B)**. Implantation of bone chips or artificial scaffolds in SCID mice can overcome this limitation **(A)** but is faced with practical limitations and the inability to support MGUS and SMM plasma cells. Additional incremental steps in humanizing NSG and NRG mice may solve this problem. A promising step in this direction is the recent development of IL-6 transgenic MIS^KI^TRG mice, which can support homing and survival of plasma cells not only from patients with frank and smoldering MM but also individuals with MGUS **(C)**.

Many advances in the field of myeloma biology, genetics, and therapy would not have been possible without preclinical investigations that relied on immunocompromised mice for hosting human myeloma cells. Human-in-mouse xenografting has a long and distinguished history in cancer, including myeloma research, beginning in the early 1960s with the discovery of the T lymphocyte-deficient nude mouse. This marked the inception of a developmental pipeline of mice that feature increasing levels of immunodeficiency. SCID (severe combined immunodeficiency) and Rag mice lack both T and B lymphocytes. The underlying genetic defects are loss-of-function mutations in *Prkdc* (protein kinase, DNA activated, catalytic polypeptide) and *Rag1* or *Rag 2* (recombination activating gene 1 or 2), respectively. Transfer of the SCID and Rag mutations on the genetic background of NOD (non-obese diabetic) led to NOD-SCID and NOD-Rag mice, which exhibit a NK (natural killer) defect on top of T and B deficiency. In addition to diminished NK cell function, NOD leads to lack of circulating complement due to deletion of the C5-encoding *Hc* gene and proclivity to Type 1 diabetes mellitus due to autoimmune insulinitis (52). Further modification of NOD-SCID and NOD-Rag mice by crossing in IL2R*γ* (interleukin-2 receptor subunit gamma) deficiency resulted in strains NSG and NRG, which are devoid of T, B and NK cells. Lack of IL2R*γ* (a.k.a. CD132 or common gamma chain, *γ*_c_) abrogates NK cells and compromises at least 6 interleukin signaling pathways: IL-2, 4, 7, 9, 15 and 21 (**Figure 3B**). NSG and NRG mice, which are commercially available and widely used in myeloma research, readily permit engraftment of human myeloma cells upon subcutaneous (SC), intravenous (IV), intraperitoneal (IP), or intratibial injection (53, 54). Strengths and limitations of myeloma xenografting have been reviewed in great depth elsewhere (55). Care must be taken to select the most appropriate HMCL for a given research question, as significant differences between cell lines exist (56, 57). 3D *in vitro* culture of myeloma cells using a variety of artificial scaffolds is an emerging technology that competes with xenografting and holds promise for drug testing for personalized myeloma therapy (58).

### Engrafting Patient-Derived Myeloma Cells in Implanted Bone Chips

Major limitations of myeloma xenografting described above include the reality that tumor growth occurs mostly outside the BM and primary patient-derived tumors do not grow at all. The latter is a fundamental flaw caused by the stringent dependency of myeloma cells on support from the human BMME. HMCLs do not exhibit this dependency because they are derived from malignant plasma cells that circulated in the peripheral blood or occurred in body cavity effusions of patients with plasma cell leukemia, the end stage of myeloma progression. The derivation from leukemic cells is also in line with the extramedullary growth pattern (plasmacytoma) of HMCL-in-mouse xenografts mentioned above. To provide a TME that is more conducive for primary myeloma cells, investigators modified SCID mice by implanting small pieces of human or rabbit bone subcutaneously. Synthetic 3D scaffolds serving as surrogate bone provide an alternative approach. These experimental model systems have come to be known as SCID-hu (12, 59–61), SCID-rab (62), and SCID-syn (14) (**Figure 3A**). They are equally capable and have greatly enhanced preclinical myeloma research (10, 11) by addressing knowledge gaps in treatment and pathophysiology: SCID-hu (63–65), SCID-rab (66–68), and SCID-syn (69, 70). However, more widespread use of these models is hampered by ethical and practical limitations, such as reliable provision of fetal human bone and difficulties in administering human myeloma cells to small implants. The fact that fetal BM does not equate with adult BM in terms of cellular composition and immune milieu, and that rabbit or synthetic bone does not fully recapitulate the myeloma-supporting properties of human bone adds an additional biological limitation. This backdrop helps explain why the use of SCID-hu, SCID-rab, and SCID-syn has been declining in recent years and why researchers have been looking for alternative strategies to propagate myeloma in laboratory mice. The most promising development to date is the humanization of NRG mice, as described below.

### Maintaining MGUS/SMM Plasma Cells in Humanized NRG Mice

Gene targeting in embryonic stem cells is a convenient research tool for substituting mouse genes with human counterparts and, thereby, humanizing laboratory mice. This approach has generated highly immunodeficient mice in which two major obstacles to engraftment of human cells have been largely overcome: innate immune rejection *via* phagocytosis and lack of activity of certain cytokines and growth factors across the human-mouse species barrier (71). Strain MIS^(KI)^TRG is an excellent example of recent developments. It features, on the genetic background of NRG, the expression of 5 human “knock in” genes encoding M-CSF (macrophage colony-stimulating factor a.k.a. colony stimulating factor 1 or CSF1), IL-3 (interleukin-3), GM-CSF (granulocyte-macrophage colony-stimulating factor a.k.a. colony stimulating factor 2 or CSF2), SIRPα (signal regulatory protein α), and thrombopoietin (72). MIS^(KI)^TRG mice exhibit improved engraftment of hematopoietic stem and progenitor cells and lend themselves to hosting PDX (patient-derived xenograft) tumors from many human cancer types. However, these mice were still unsuitable for stable engraftment of primary myeloma cells. Cognizant of the critical role of IL-6 for growth and survival of neoplastic plasma cells (73), Madhav Dhodapkar and his associates added a human IL-6 allele to strain MIS^(KI)^TRG, thus generating IL-6 transgenic MIS^(KI)^TRG, or MIS^(KI)^TRG6 mice (**Figure 3C**) (74). The introduction of human IL-6 resulted in a remarkable breakthrough for preclinical myeloma research, because for the first time it allowed engraftment and propagation of primary MM cells in a reliable and reproducible fashion. What is more, MIS^(KI)^TRG6 supported engraftment of SMM and MGUS plasma cells upon transfer of CD3-depleted BM mononuclear cells to recipient bone. The finding that, unlike NDMM cells, RRMM cells actively homed to and expanded in other sites of the skeleton resembled the more advanced tumor progression stage of relapsed compared to new myeloma. Finally, while RRMM remained confined to bone, tumor samples from patients with plasma cell leukemia demonstrated the kind of systemic, extramedullary dissemination pattern that one might expect from a leukemic cell clone.

### Utility of Myeloma Xenografting in Immunotherapy Research

Of the three principal model systems of myeloma xenografting described above, the HMCL-in-mouse approach, has probably had the greatest impact on immunotherapy research in myeloma. While MIS^(KI)^TRG6 mice have not yet been used to that end, the implantation-based SCID models have largely been supplanted by HMCL-in-mouse xenografts, as mentioned above. Indeed, HMCL xenografting appears to lend itself readily to the complex requirements of experimental immunotherapy. Often this involves adoptive immune cell transfer to study mice and testing of new therapeutic antibodies or antibody-drug conjugates (ADCs) in mice co-treated with established myeloma drugs or candidate small-drug inhibitors. HMCL xenografts are often used as a first choice when the efficacy of cytotoxic T cells, NK cells, or engineered killer cells to remove myeloma in an intact organism *in vivo* is to be evaluated. To highlight but a few examples of their utility, HMCL xenografts have majorly contributed to the development of CAR-T treatments for BCMA (75) and newly emerging CAR-T targets such as CD229 (SLAMF3, LY9) (76). HMCL xenografts have been successfully employed to assess a monoclonal antibody to transferrin receptor 1, a newly emerging molecular target expressed on the surface of myeloma cells (77, 78). Similarly, HMCL xenografts have been used to evaluate AMG 701, a half-life extended BITE that binds to BCMA on myeloma cells and CD3 on T cells (79), and to demonstrate that the therapeutic efficacy of daratumumab in myeloma may be enhanced when CD38 in NK cells has been deleted (80). This body of work illustrates the rapid evolution of the myeloma immunotherapy landscape and that HMCL xenografting is poised to add further value to the field as we go forward.

## Autograft Models Of Myeloma

### 5TMM

**Figure 4:**
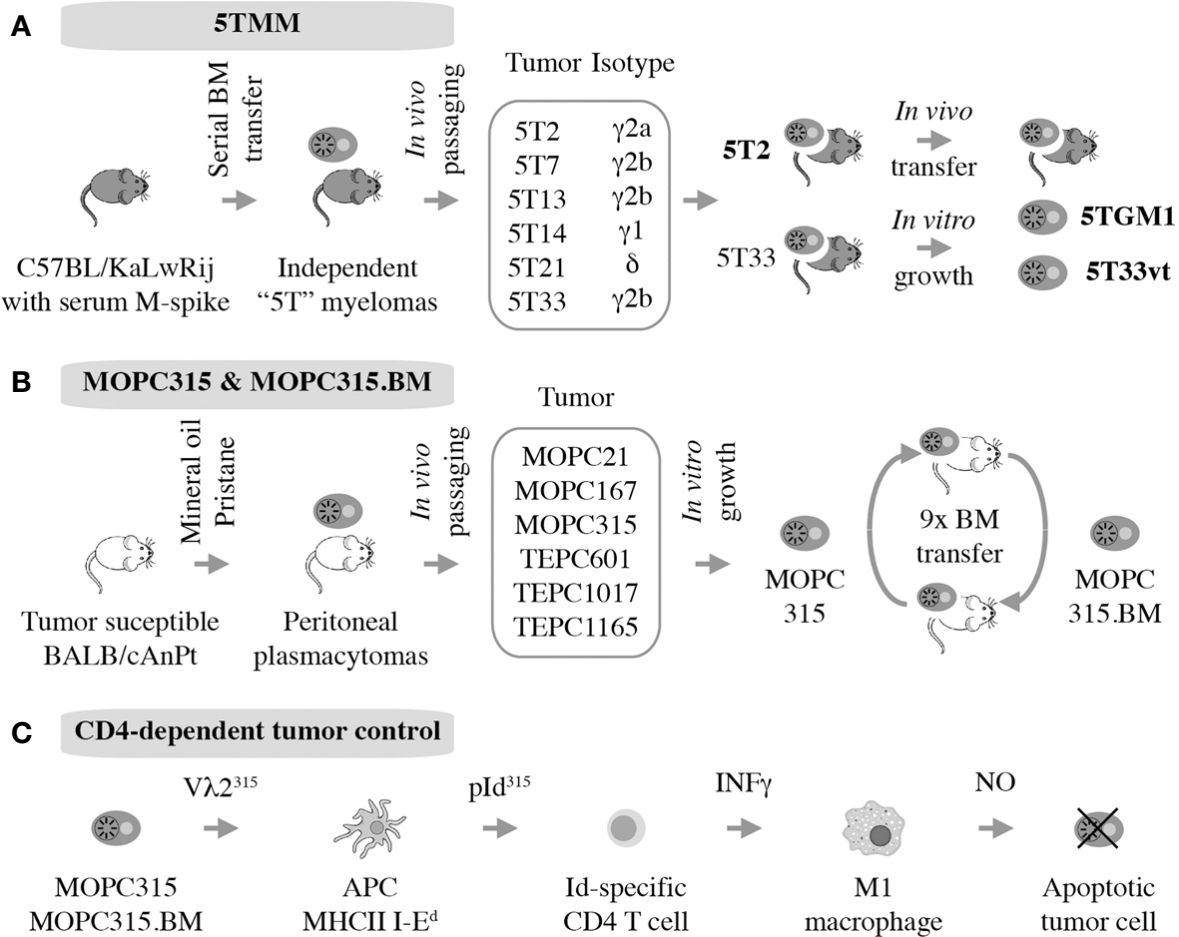
Autografting mouse myeloma in immunocompetent mice. Two models have been established. 5TMM is on a genetic background that is highly similar to B6 and includes two continuous cell lines, 5TGM1 and 5T33vt, that are widely used **(A)**. MOPC315 is a peritoneal plasmacytoma on the genetic background of C that has given rise to a BM-seeking subline, MOPC315.BM, that holds great value for myeloma immunology research. **(B)** Decades of research by Bogen and colleagues have shown indirect CD4+ T cells mediated killing via interactions with cytotoxic macrophages, further demonstrating the utility of MOPC315.BM as an immunological research tool **(C)**.

The 5T mouse model of human multiple myeloma, or 5TMM for short, is a versatile research tool for fundamental and applied studies on plasma cell malignancies. The model is based on the genetic proclivity of inbred C57BL/KaLwRij mice (closely related to the commonly used C57BL/6) (81) to spontaneously develop a benign monoclonal gammopathy (serum paraprotein) or MGUS-like condition (82) that can progress to frank myeloma (15, 83). Using serial *in vivo* propagation of bone marrow cells from independent C57BL/KaLwRij donors containing different paraproteins, a number of transplantable myeloma-like plasma cell tumors were generated (**Figure 4A**). Two of these, dubbed 5T2 and 5T33, were fully established and generously shared with qualified investigators in Europe, the United States, and elsewhere. 5TMM tumors cause osteolytic lesions (83) and recapitulate other features of human myeloma bone disease (84, 85). 5TMM tumors grow in a fully immunocompetent BMME and are easily tracked *in vivo* using radiological methods including X-ray and PET imaging (86). Unlike 5T2, which can only be maintained by passaging from mouse to mouse, two continuous cell lines were derived from 5T33: 5TGM1 (87, 88) and 5T33vt (89). The genetic differences between these cell lines and 5T2 have been recently determined using NGS (90). This revealed an additional strength of 5TMM in regard to modeling human myeloma; i.e., the significant overlap of patterns of somatic mutations across the human-mouse species barrier, particularly with respect to copy number changes of genes involved in gain of 1q and deletion of 13q in human myeloma (90). The availability of cell lines, 5TGM1 and 5T33vt, has greatly enhanced the flexibility and impact of the 5TMM model. For example, the cells can be easily modified by virtue of enforced up or down regulation of genes of interest or the introduction of reporter genes for whole-body fluorescence or bioluminescence imaging of tumor growth in a quantitative, objective manner (91). The cell lines have also facilitated the examination of myeloma-immune interactions *in vitro* and *in vivo*. For example, an early study using 5TGM1 demonstrated that myeloma cells inhibit the differentiation of BM-derived dendritic cells (DCs) and interfere with their function to induce cytotoxic and humeral immune responses (92). This is relevant for ongoing efforts in human myeloma to use DCs for vaccination approaches aimed at eliciting a robust T cell-dependent cytotoxic immu response (93).

### 5TMM as Research Tool for Immune Regulation of Myeloma

The broad utility of 5TMM for studies on the immunopathology and -therapy of myeloma was recognized soon after the first tumors were established. Initial investigations described the role of the paraprotein idiotype (Id) in immune regulation (94) and immune therapy (95) of plasma cell neoplasia. Follow-up studies relying on 5TGM1 demonstrated that the idiotype is a myeloma-specific antigen that can induce an Id-specific cytotoxic T cell (CTL), T helper 1 (Th1) and T helper 2 (Th2) response (96). CTLs and Th1s were found to suppress myeloma growth, whereas Id-specific Th2 cells promoted it (96) – a preclinical clue in support of the contention that modulation of the Th1:Th2 axis might have therapeutic benefits in myeloma. The finding that 5T-bearing mice exhibit an increase in the ratio of regulatory T cells (Tregs) to T effector cells (97) proved relevant for human myeloma when it became clear that patients with new disease contain elevated numbers of Tregs in the peripheral blood (98). With respect to myeloma immunotherapy, the 5T33 model made conceptual contributions to developing DC-based MM vaccines for idiotype protein (99). This included the design of more effective adjuvants based on CpG and IFN-*α* (100) and the realization that myeloma cell lysates provide a more powerful DC vaccine than idiotype protein and adjuvant, alone (101). These advances were confirmed in a clinical study a few years ago showing that a patient-derived DC-MM cell fusion (hybridoma) vaccine improved the therapeutic response in a quarter of myeloma patients post-ASCT from partial to (nearly) complete (102). 5TMM has also been used to examine immunomodulatory myeloma treatments at the preclinical level; e.g., investigators demonstrated that CD4 T cells were vital for lenalidomide’s activity, while NK, B or CD8 T cells were not (103). Activation of innate-like invariant natural killer T (iNKT) cells, a cell type that has not yet been extensively examined in human myeloma, led to significantly increased survival of 5T33-bearing mice (104). The 5T33 model has also contributed early on to the CAR-T therapy field by showing that treatment using NKG2D-targeted CAR-T cells prolonged survival of tumor-bearing mice and induced a tumor-specific memory response (105). Furthermore, 5T33 not only demonstrated efficacy of immune checkpoint inhibitor (CPI) therapy using antibody to programmed death receptor-1 (PD-1) or its ligand (PDL-1), but also showed that CD8^+^ T cells in tumor-bearing mice post-ASCT significantly upregulated PD-1 (106, 107). In summary, the practical limitation to *in vivo* studies using 5TMM requiring the genetic background of C57BL/KaLwRij (108) is a small inconvenience compared to the potential contribution of this model to aiding immunotherapy development for patients with myeloma.

### MOPC315.BM

MOPC315 is an IgA-producing peritoneal plasmacytoma (PCT) that arose half a century ago (109) in a BALB/c (C) mouse treated with intraperitoneal injections of mineral oil (110). MOPC315 has been used for decades in studies on immune regulation of malignant plasma cell growth (111, 112) although it is not representative of human MM, which grows in the hematopoietic BM and depends on the BMME for survival. In a major step forward, this shortcoming was remedied by the development of a subline of MOPC315, dubbed MOPC315.BM, generated by serial IV autografting of BM-derived plasma cells for nine generations. In the course of *in vivo* propagation, tumor cell variants with exquisite affinity to the BM increased oncogenic potency (~1 month median survival of tumor-bearing mice), and capacity for BM homing and bone destruction were preferentially selected (**Figure 4B**). Transfection with a luciferase reporter further increased the utility the cell line (16). MOPC315.BM is now increasingly used in preclinical myeloma research. For example, it provided the foundation for recent studies on the involvement of IL-34 and notch signaling in the pathophysiology of the focal and systemic bone loss in mice that mimics human myeloma bone disease (MBD) (113, 114). Additionally, MOPC315.BM has been employed to demonstrate that eosinophils and megakaryocytes support malignant plasma cell growth in the BM (115) and that oncolytic myxoma virus, in conjunction with ASCT, may be an effective treatment for PCT-bearing mice (116). Importantly, together with its parental line, MOPC315.BM has made major contributions to our appreciation of CD4 T cell responses in immunosurveillance and -therapy of myeloma (117), briefly summarized below.

### Lesson on CD4-Dependent Control of Malignant Plasma Cell Growth

Strong evidence supports the significance of Id-specific CD4 T cells in clearance of MOPC315 plasma cells *in vivo* (118), confirming matching results in the 5TMM model described above. In a remarkable continuity of investigations that span more than 25 years, Bjarne Bogen and his colleagues have unequivocally shown that tumor cell-produced monoclonal Ig gives rise to a tumor-specific antigen (TSA) in the MOPC315 model system. The antigen is processed by professional antigen-presenting cells (APCs) that include tumor-infiltrating macrophages in the subcutaneous MOPC315 model and BM macrophages in the medullary MOPC315.BM model (119). With help of MHC class II-encoded I-E^d^ surface protein, APCs present the antigen to CD4 Th1 cells as a λ2 light-chain V region-derived idiotypic (Id) peptide; i.e., a neoepitope. This results in Th1-dependent production of IFN-*γ*, which activates bystander macrophages and promotes their polarization to the tumoricidal M1 phenotype. In turn, M1 macrophages upregulate inducible nitric oxide synthetase (iNOS) to produce and release nitric oxide (NO) into the extracellular milieu. NO then kills neighboring tumor cells using a mechanism that involves reactive nitrogen species (e.g., peroxynitrite) and activates the intrinsic pathway of programmed cell death (apoptosis). Thus, in the MOPC315 model system, CD4^+^ T cells kill tumor cells indirectly with the assistance of cytotoxic macrophages (**Figure 4C**). MOPC315.BM has provided additional insights into myeloma immunology. Examples include the interaction of myeloid-derived suppressor cells and T cells *in vivo* (120), the development of allogeneic T cell treatments for myeloma that may circumvent GvHD (121, 122), and the evaluation of novel DNA vaccines for immunotherapeutic purposes (123). Similar to the inconvenience of the genetic background of 5TMM, MOPC315.BM is on the genetic background of BALB/c (C), which is not as widely used in cancer immunology as B6. However, this is a small price to pay considering the value MOPC315.BM can add to the immune revolution in myeloma treatment (124).

## Spontaneous Plasma Cell Tumors In Laboratory Mice

### Inducible, Inflammation-Dependent Peritoneal Plasmacytoma

MOPC315 is but one representative of a large panel of peritoneal plasmacytomas that has been developed single handedly in the 1960s and 1970s by Dr. Michael Potter at the US National Cancer Institute, Bethesda, Maryland. He discovered that IP treatment of C mice using certain mineral oils (110) or a chemically defined component thereof, called pristane (2,6,10,14-tetramethylpentadecane) (125), induced development of MOPC (mineral oil induced plasmacytoma) and TEPC (tetramethylpentadecane induced plasmacytoma) tumors, respectively. Plasmacytomas induced in this fashion were crucial for basic research breakthroughs in antibody structure (126) and monoclonal antibody (hybridoma) technology, which began with MOPC21 (127). Unlike most inbred strains of mice, C is highly susceptible to plasmacytoma (128) due to a complex genetic trait that includes hypomorphic (weak efficiency) alleles of genes that encode the cell cycle inhibitor p16^INK4a^ (129) and the FKBP12 rapamycin-associated protein Frap (130). Virtually all peritoneal PCTs harbor a *Myc*-activating chromosomal translocation (131) that takes the form of a balanced T(12;15)(*Igh-Myc*) in the majority (~85%) of cases. Plasmacytoma induction requires maintenance of mice in a non-SPF (specific pathogen-free) environment rich in antigenic stimuli including gut flora-derived antigens (132). Consistent with that, C mice raised under SPF or germ-free conditions exhibit a dramatically reduced tumor incidence (133) or fail to develop plasmacytoma altogether (134). Peritoneal plasmacytoma is the premier mouse model of inflammation-induced extramedullary myeloma has been used for decades to learn about immune regulation of malignant plasma cell growth (111, 112). However, BALB/c plasmacytomas are not widely used in myeloma research today because more accurate, transgenic mouse models of the disease have become available. These will be described in the following section.

### Transgenic Mouse Models of Human Myeloma and Related Plasma Cell Neoplasms

**Figure 5:**
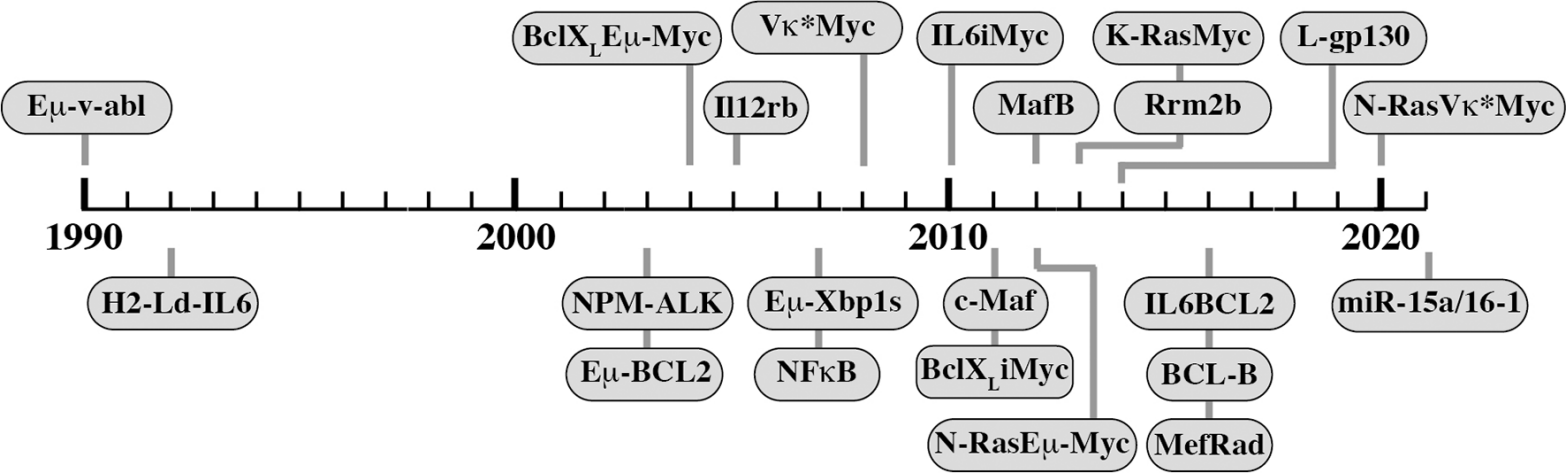
Transgenic mouse models of human plasma cell myeloma and extramedullary plasmacytoma. Shown is a timeline of model development that begins with Eµ-v-abl developed by Susan Cory’s group at WEHI and published in 1990. The H2-L^d^-IL6 model of human plasmacytoma published in 2002 gave rise to the double-transgenic MycIL6 and BCL2IL6 models that take advantage of oncogene collaboration to accelerate neoplastic plasma cell development. Similarly, Vκ*MYC, the premier model of myeloma immunology research, was recently accelerated by breeding in a mutated *Ras* gene, leading to the highly promising VQ model published in 2020.

Genetic modification of the mouse germline has been employed by several independent research groups to generate transgenic strains of mice that are prone to spontaneous plasma cell tumors (PCT) that replicate important features of human MM. Mice of this sort exhibit a predictable progression pattern from MGUS- and SMM-like precursor conditions to frank plasma cell neoplasia. This pattern is key for preclinical assessments of myeloma preventions, a hot topic in clinical myeloma research (135). PCT-prone mice feature a fully intact innate and adaptive immune system that is likely to adapt to tumor development much the same way as the human immune system adapts to myeloma (**Figure 2A**). Hence, trialing newly developed immunotherapeutics in mice that are genetically susceptible to PCT is poised to yield more complete and higher-quality information than one might get from mice that are immunocompetent but not undergoing tumorigenesis (136). The same argument can be made for the preclinical testing of complex treatment regimens that combine HSC transplantation and established myeloma drugs (PIs, IMiDs) with novel immunotherapies and small-compound inhibitors. Evaluating these types of treatment in PCT-susceptible mice may more closely mimic the response of myeloma patients exposed to triplet and quadruplet drug regimens (137). **Figure 5** presents a developmental timeline of genetically engineered mouse models (GEMMs) of human myeloma and related malignancies. **Table 1** provides details on tumor incidence and phenotype, genetic drivers of tumor development, and genetic background of mice. An exhaustive discussion of individual models is beyond the scope of this review. To that end, the reader is referred to the primary literature and outstanding recent reviews from Tassone (55), Vlummens (138) and their associates. A general rule that may be gleaned from the table is that single-transgenic models exhibit delayed tumor onset and a relatively low tumor incidence. To accelerate plasma cell neoplasia, investigators have taken advantage of oncogene collaboration in double-transgenic mice, such as IL6Myc (139) and Bcl-X_L_iMyc (140), that exhibit short tumor onset and full penetrance of the malignant phenotype (100% tumor incidence). Inducible transgenes such as L-gp130 (141) and models based on adoptive transfer of genetically modified B cells (142–144) serve the same purpose; i.e., faster and more consistent tumor development. Importantly, Vκ*MYC, developed by Marta Chesi and Leif Bergsagel at Mayo and generously shared with investigators in many countries, is the only model at this juncture for which robust immunology work is available. This is one of several reasons why Vκ*MYC is widely considered in the myeloma community as the gold standard of mouse models. Advances in immunosurveillance and immunotherapy of myeloma made possible by Vκ*MYC will be briefly discussed below.

**Table 1:** Transgenic mice prone to spontaneous plasma cell tumors recapitulating hallmarks of human plasma cell neoplasms including multiple myeloma.

Row	Mouse model ^1^	TG ^2^	Back-ground ^3^	Year ^4^	Ref. ^5^	Survival of mice ^6^	Percent tumors ^7^	Tumor phenotype ^8^
1	Eµ-v-abl	1	B6	1990	(160, 161)	>1 year	60	PC
2	H2-Ld-IL-6	1	C	1992	(162, 163)	250 days	60	PC > Ly
3	NPM-ALK	1	B6, C	2003	(164)	18 weeks	100	PC
4	Eµ−BCL2	1	B6, C	2003	(165)	120 days	100	PC > Ly
5	BclX_L_EµMyc	2	Mixed ^16^	2004	(166)	50 days	100	PC > MM
6	Eμ-Xbp1s	1	B6	2007	(167)	2 years	20	MM > PC
7	NFκB	1	Mixed ^17^	2007	(168, 169)	50 weeks	80	PC > Ly
8	Vk*Myc	1	B6 ^18^	2008	(170)	660 days	100	MM
9	Il12rb	1	B6	2005	(171)	2 years	30	PC
4	IL6Myc ^9^	2	C	2010	(136, 172)	12 weeks	100	PC > MM
10	c-MAF	1	B6	2011	(173)	>2 years	30	Ly > PC
11	BclX_L_iMyc ^10^	2	Mixed ^19^	2011	(174)	135 days	100	PC > MM
12	N-RasEµMyc ^11^	1	Mixed ^16^	2012	(175)	75 days	100	Ly > PC
13	MafB	1	B6	2012	(176)	1 year	45	MM > PC
14	Rrm2b	1	B6	2013	(177)	30 days	30	PC
15	K-RasMyc ^12^	2	C	2013	(139, 140)	50 days	100	PC
16	L-gp130 ^12^	1	B6	2014	(178)	200 days	100	MM
17	IL6BCL2 ^9,12,13^	2	C	2016	(141)	5 months	100	MM > PC
18	BCL-B	1	B6	2016	(179)	500 days	100	MM
19	MefRad	2	B6	2016	(180)	480 days	70	MM > PC
20	L-gp130 ^14^	1	B6	2019	(138)	5 months	50	MM
21	N-RasVk*Myc ^15^	2	B6	2020	(181)	350 days	60	MM
22	miR15a/16-1 ^16^	2	B6	2021	(182)	>1 year	45	Ly > PC

^1^ Mouse models in chronological order of development, as shown in **Figure 5**.

^2^ Mouse models rely on one transgene or two transgenes to drive tumor development.

^3^ Genetic background of mice is either C57BL/6 (B6), BALB/c (C) or mixed.

^4^ Publication year.

^5^ Original reference. A follow-up publication is included in some cases to provide more complete information on survival, tumor incidence, and tumor phenotypes.

^6^ Median, mean, or estimated survival of mice, depending on results available. Surrogate of tumor onset.

^7^ Percent tumor incidence (rounded).

^8^ Phenotypes include plasma cell myeloma (MM), plasmacytoma (PC), and B lymphoma (Ly). The latter often exhibits plasmablastic features. The preponderance of a particular phenotype is indicated by a “larger than” symbol for models yielding different phenotypes.

^9^ Using the same IL6 transgene as in row 2.

^10^ Using the same Bcl-X_L_ encoding BCL2L1 transgene as in row 5.

^11^ Using the same Myc transgene as in row 5.

^12^ Model that relies on adoptive transfer of genetically modified B-lymphocytes to a preconditioned host in which neoplastic plasma cell development takes place.

^13^ Using the same BCL2 transgene as in row 4.

^14^ Using an inducible version of the transgene from row 16.

^15^ Using the same Myc transgene as in row 8.

^16^ Loss of microRNA in germinal center B cells effected by transgenic, AID-dependent Cre recombinase.

^17^ (B6 x FVB/N) F1 hybrids

^18^ B6 and SJL alleles.

^19^ Transfer of Vκ*Myc onto background of C abolished cancer phenotype (183).

^20^ B6, 129SvJ and FVB/N alleles.

### Advances in Myeloma Immunology Made Possible by Vκ*MYC

**Figure 6:**
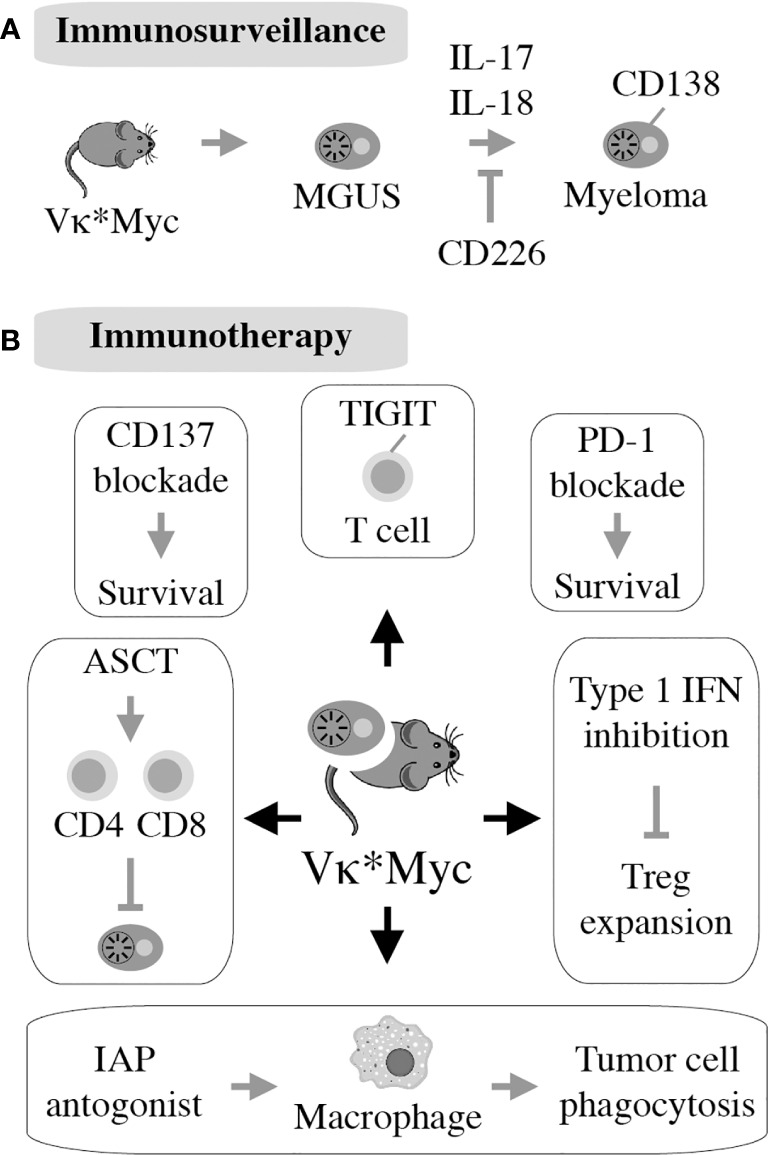
First described in 2008, the Vk*MYC model takes advantage of AID-activated MYC to induce myeloma on the B6 background. All 122 mice in the original study had monoclonal plasma cell expansion in the BM resembling human MM. Eighty percent of mice had measurable M-spike by 50 weeks of age. Additionally, aged Vk*MYC mice displayed many hallmarks of human MM, including bone loss and protein deposition in the kidneys (150). This allows for thorough studies of the MGUS to MM transition in this model **(A)**. Vk*MYC mice accurately predict clinical efficacy of myeloma drugs (151) and provide a good model for experimental oncolytic immunotherapy (152) **(B)**.

The realization that Vκ*MYC-dependent myeloma causes changes in immune regulation in mice comparable to changes seen in patients with myeloma (145) laid the foundation for mechanistic studies describing role of specific pathways of immunity to Vκ*MYC-driven tumor development. The first investigation along this line revealed the importance of CD226 for immune surveillance of myeloma. Lack of CD226 reduced the anti-myeloma response of NK and CD8 T cells, resulting in quicker tumor progression and decreased overall survival of Vκ*MYC mice (146). Another insight afforded by Vκ*MYC concerned the intriguing link between microbial gut flora and IL-17-driven tumor progression. The underlying mechanism is complex but involves an increase in Th17 cells and activation of eosinophils. Not coincidentally, therapeutic control of these changes using antibodies to IL-17 and IL-5 delayed tumor progression (147). Vκ*MYC also provided definitive genetic evidence on the involvement of the pro-inflammatory cytokine IL-18 in myeloma progression (148). This was attributable to IL-18-dependent generation of myeloid-derived suppressor cells (MDSCs), an important driver of the dysfunctional immune environment in human myeloma (**Figure 2B**). Another study demonstrated that tumor progression and dissemination in Vκ*MYC is not under exclusive control of the TME. Insead, it is regulated, in part, by properties of tumor cells such as expression of CD138 (149) (**Figure 6A**). Vκ*MYC has also impacted the field of myeloma immunotherapy in more ways than one. One study showed that treatment of mice using IAP (inhibitor of apoptosis) antagonists activated an acute inflammatory response that led to enhanced tumor phagocytosis by macrophages. Interestingly, co-treatment using antibody to PD1 led to an additional increase in survival of mice (153). By implicating the upregulation of TIGIT (T-cell immunoglobulin and ITIM domain) on CD8 T cells, Vk*MYC has also contributed to our understanding of T cell exhaustion in myeloma. Checkpoint blockade of TIGIT prolonged survival of mice and reduced levels of immunosuppressive IL-10 produced by dendritic cells (154–156). Finally, studies using Vk*MYC demonstrated that: blocking type 1 interferon signaling may inhibit Treg expansion in myeloma (157), antibody to CD137 holds promise as a consolidation treatment in myeloma (158), and HSC transplantion may facilitate both a robust anti-MM CD8 T cell response and a myeloma-specific T cell memory (159) (**Figure 6B**). The impressive body of work summarized above strongly suggests that Vk*MYC provides a valuable blueprint for immunological studies using other mouse myeloma models included in **Table 1**.

## Research Gaps And Future Directions

### Following in the Footsteps of Vκ*MYC

One line of future investigation should be aimed at determining whether the immune changes seen in tumor bearing Vk*MYC mice also occur in other strains of mice prone to spontaneous PCT (**Table 1**). Independent confirmation would lend support to the contention that the observed changes represent bona fide biological sequelae of neoplastic plasma cell development rather than a special feature of this particular model. Uncovering significant differences in immune cell compartments or pathways of immunity between different mouse models may also be of value because it may help investigators match a particular type of human myeloma in terms of progression stage (e.g., NDMM vs RRMM), outcome risk (e.g., standard *vs* high risk), or molecular subgroup (184) with the most appropriate mouse counterpart. Since human myeloma exhibits a great deal of diversity in cytogenetic, gene expression, epigenetic, immunologic, and other features (185), MM should be represented by a collection of models that mirror that diversity. However, this goal has not yet been achieved as the models listed in **Table 1** represent but a narrow snapshot of the human myeloma landscape. With the exception of the most recently developed model that relies on a Cre recombinase effected loss of the microRNA-encoding tumor suppressor locus, *miR-15a/16-1*, malignant development is driven in these models by a limited set of oncogenes on the uniform, homogeneous genetic background of inbred mice (186). The overrepresentation of Myc, IL-6, and Bcl-2 family genes, particularly among the more thoroughly investigated models, underscores the narrowness and redundancy of the present situation.

Be this as it may, Vk*MYC and related models stand ready both to shed light on long-standing questions in myelomagenesis, such as the role of antigen and germinal center reentry of tumor precursors (187) and to revisit difficult issues in myeloma immunotherapy, such as the benefits of immune checkpoint inhibition (CPI) (188), which remain unclear at this juncture (189). Since Vk*MYC mice undergoing IAP inhibition responded to CPI with increased survival (153), in-depth analysis of that response may provide clues for how to incorporate CPI in human myeloma treatment protocols. Vk*MYC and other transgenics may also assist in validating novel immunotherapies that are emerging from exploratory studies using transplantation-based mouse models. Two recent advances that relied on 5TMM and HMCL, respectively, concerned the combination of vaccination and epigenetic therapies (190) and a neat strategy for enhancing the efficacy of daratumumab by genetic engineering of NK cells (191). Considering the increased interest of the clinical myeloma community in tumor prevention (192), Vk*MYC and related models may also afford opportunities for the preclinical evaluation of candidate interventions to block the progression of high-risk MGUS and SMM to frank myeloma.

### Toward a Robust PDX Model of MM

Despite the breakthrough accomplishments of the MIS^(KI)^TRG6 mouse described above, this xenograft model of human myeloma is still limited compared to well-established PDX (patient derived xenograft) models of solid cancer (193) and emerging PDX models of lymphoma (194). Biological limitations of MIS^(KI)^TRG6 and the parental strain, MIS^(KI)^TRG, include proclivity to anemia and quick exhaustion of human grafts after cell or tissue transfer (195). There are also some thorny non-biological limitations, including intellectual property rights that have prevented the wider distribution of the MIS^(KI)^TRG6 thus far. Hence, additional work is warranted to improve upon this model and develop humanized laboratory mice that lend themselves to the preclinical evaluation of myeloma immunotherapy and precision medicine approaches. To that end, a fundamental conceptual consideration is the recognition that increasing levels of immunodeficiency result in better engraftment of tumor cells (**Figure 3**) but diminished opportunities to assess the impact of the immune system on myeloma biology and treatment responses. One way to address this conundrum is a non-genetic form of humanizing mice by means of adoptive transfer of human hematopoietic stem and progenitor, T, NK, and other blood cells. All of these cells are easily obtained from patients with myeloma, particularly those undergoing SCT, and can be engrafted in mice together with BM-derived malignant plasma cells. Disadvantages of this approach include the small experimental window in the adoptively transferred mouse (on the order of a few days) and the high risk of GvHD that may distort study results (196).

A parallel way forward is to continue with the genetic humanization of laboratory mice. The aim is to generate humanized mouse PDX myeloma models that will be as promising for immunotherapy research as the new generation of mouse PDX carcinoma models is (197, 198). Molecular targets of humanization include components of the HSC niche (e.g., c-kit and Flt3) and biological pathways of myeloid and NK reconstitution (e.g., c-kit ligand and GM-CSF). Additional targets include the major histocompatibility complex (e.g., deletion of mouse beta-2 microglobulin) and, importantly, immune checkpoints such as CTLA-4, the CD47 “don’t eat me” signal to macrophages, BTLA (CD272), TIM3, GITR, OX40 and others (199, 200). The long list of checkpoint genes underscores the elusiveness of humanizing the immune response of mice completely. The development of specialized, partially humanized mouse models dedicated to specific aspects of immunotherapy is therefore a viable compromise and a step in the right direction. A good example along this line is the generation of mice that contain a humanized form of cereblon (201), the molecular target of IMIDs. It renders the mice responsive to drugs of this sort, which are not active in normal mice. Three additional examples of humanized mouse models relevant for myeloma research are NOG-hIL-6 (202), B6-hCD3E (170) and B6-hTIGIT (203), which facilitate preclinical studies on MDSCs, BITEs and CPI, respectively.

## Conclusion

Although immunotherapy holds great promise for revolutionizing myeloma treatment (124), much remains to be learned. Currently, only a fraction of patients achieves a complete, long-lasting treatment response and a functional or definitive cure remains elusive for the great majority of patients. Accurate mouse models of myeloma are needed to close current knowledge gaps and accelerate the design and testing of new immunotherapies. The workhorses of preclinical myeloma research, HMCL-in-mouse xenografting and mouse-in-mouse autografting, will continue to be employed to that end, but we anticipate that humanized PDX models of myeloma (204) and transgenic mouse models of myeloma will become more important as we go forward. These models may elucidate the complex mechanisms underlying myeloma immunopathology and -therapy and minimize the risk of failure in challenging and expensive clinical trials. Sharing experimental model systems without strings attached, enhancing collaboration among regional core facilities and national reference centers, and establishing standards for high scientific rigor for the preclinical myeloma research will contribute to a future for patients with myeloma that is hopeful and bright.

## Author Contributions

MP performed the primary literature review and prepared the table. SJ edited the text, prepared the figures and formatted the final version. All authors contributed to the article and approved the submitted version.

## Funding

This work was supported by the William G. Schuett, Jr., Multiple Myeloma Research Endowment. Additional support was provided by NCI R01CA204231 (to SR), NCI R01CA236814 and DOD CA180190 (to FZ) and NCI R01 CA151354 (to SJ).

## Conflict of Interest

The authors declare that the research was conducted in the absence of any commercial or financial relationships that could be construed as a potential conflict of interest.

## Abbreviations

BM, bone marrow; BMME, bone marrow microenvironment; B6, C57BL/6; C, BALB/c; Ig, immunoglobulin; IMiD, immunomodulatory drug; IP, intraperitoneal; IV, intravenous; mAb, monoclonal antibody; MGUS, monoclonal gammopathy of undetermined significance; MM, multiple myeloma; NDMM, newly diagnosed multiple myeloma; PI, proteasome inhibitor; RRMM, relapsed/refractory multiple myeloma; SC, subcutaneous; SMM, smoldering multiple myeloma; TME, tumor microenvironment.
